# Quality of focused thoracic ultrasound performed by emergency medical technicians and paramedics in a prehospital setting: a feasibility study

**DOI:** 10.1186/s13049-021-00856-8

**Published:** 2021-02-25

**Authors:** Pia Iben Pietersen, Søren Mikkelsen, Annmarie T. Lassen, Simon Helmerik, Gitte Jørgensen, Giti Nadim, Helle Marie Christensen, Daniel Wittrock, Christian B. Laursen

**Affiliations:** 1 Odense Respiratory Research Unit (ODIN), Department of Clinical Research, University of Southern Denmark, Sdr. Boulevard 29, entrance 87, 1st floor, 5000 Odense C, Denmark; 2 Regional Center for Technical Simulation, Odense University Hospital, 5000 Odense C, Denmark; 3 The Prehospital Research Unit, Region of Southern Denmark, Odense University Hospital, 5000 Odense C, Denmark; 4 Department of Emergency Medicine, Odense University Hospital, 5000 Odense C, Denmark; 5 Department of Quality & Education, Ambulance Syd, Region of Southern Denmark, 5220 Odense SØ, Denmark; 6 Ambulance Syd, Region of Southern Denmark, 5220 Odense SØ, Denmark; 7 Department of Clinical Research, University of Southern Denmark, 5000 Odense C, Denmark; 8 Department of Respiratory Medicine, Odense University Hospital, 5000 Odense C, Denmark

**Keywords:** Thoracic ultrasound, Respiratory symptoms, Prehospital ultrasound

## Abstract

**Background:**

In a prehospital setting, the severity of respiratory symptoms in patients calling for an ambulance differ. The initial evaluation, diagnosing, and thereby management can be challenging because respiratory symptoms can be caused by disease in many organs. Ultrasound examinations can contribute with important information and support the clinical decision-making. However, ultrasound is user-dependent and requires sufficient knowledge and training. The aim of this study was to explore the quality of thoracic ultrasound examinations performed on patients by emergency medical technicians and paramedics in a prehospital, clinical setting.

**Methods:**

From November 2018 – April 2020, Danish emergency medical technicians and paramedics (*n* = 100) performed thoracic ultrasound examinations on patients with respiratory symptoms using a portable ultrasound device. The ultrasound examinations were stored and retrospectively assessed by a reviewer blinded to the patients’ symptoms and history, as well as the emergency medical technicians’ and paramedics’ findings. The image quality was scored from 1 to 5. The findings determined by the reviewer was then correlated with a questionnaire filled out by the emergency medical technicians and paramedics regarding ultrasonic findings and potential change in treatment or management of the patient. The agreement in percentage and as Cohen’s kappa was explored.

**Results:**

A total of 590 ultrasound examinations were assessed, resulting in a median image quality score of 3 (IQ1 = 4, IQ3 = 3). The overall agreement in percentage between the emergency medical technicians and paramedics and reviewer was high (87.7% for a normal scan, 89.9% for interstitial syndrome, 97.3% for possible pneumothorax, and 96.3% for pleural effusion). Cohen’s kappa varied from 0.01 for possible pneumothorax to 0.69 for pleural effusion. Based on the questionnaires (*n* = 406), the ultrasound examination entailed a change in treatment or visitation in 48 cases (11.7%) which in this study population encompasses a number-needed-to-scan of 8.5.

**Conclusion:**

Emergency medical technicians and paramedics perform focused thoracic ultrasound examinations with adequate image quality sufficient to determine if pathology is present or not. The emergency medical technicians’ and paramedics’ assessment correlates to some extent with an experienced reviewer and their findings are most reliable for the inclusion of a normal scan or inclusion of pleural effusion. Implementation could possibly impact the number of patients receiving correct prehospital treatment and optimal choice of receiving facility.

## Introduction

Cardiac arrest, severe trauma, chest pain, stroke, and respiratory difficulties are defined as the “First Hour Quintet” [1]. These five conditions are characterised by being life-threatening. Rapid evaluation is essential to begin fast goal-directed treatment and to decrease the morbidity and mortality. A wide range of pulmonary diseases can cause acute respiratory failure, but also other organ systems, e.g., heart failure, renal failure, or septic shock can present with respiratory symptoms –which makes it challenging to identify the cause of the symptoms [2, 3]. In a prehospital setting, the diagnostic resources are limited and need to function in more extreme conditions (e.g. darkness, rain, high and low temperature) compared to those found in an in-hospital setting. Thoracic ultrasound has shown high sensitivity and specificity for many common causes of respiratory symptoms and thereby, ultrasound can support the clinical decision-making and benefit the patients [4–6].

Correct prehospital management of patients with respiratory symptoms is essential to increase survival [2]. The Emergency Medical Technicians (EMTs) and paramedics (PMs) are in charge of the management including initial evaluation, treatment, and making decisions regarding time spend on the scene, transport, and location [7]. Most often, the decisions are based on patient history, vital signs, and few objective examinations like auscultation [6, 8, 9]. Making ultrasound available for the EMTs and PMs creates a new option for assessment of the patient. Ultrasound has previously been a diagnostic tool reserved for physicians, but within the last couple of years different healthcare professions have started to use it in a broad variation; e.g. as guidance for peripheral vein catheter insertion [10], for tele-echocardiography of chronic heart failure patients [11], or as guidance for physiotherapeutic training of diaphragm and thorax in the intensive care unit [12].

However, diagnostic ultrasound examinations are operator-dependent, and to reach a sufficient and high diagnostic accuracy, correct technical execution of the ultrasound examination, as well as correct image interpretation are prerequisites [13]. Furthermore, the operator must possess the capability to integrate the ultrasound findings in context to the patient’s history, symptoms, and other clinical parameters. An additional challenge is the need for the ultrasound equipment to function regardless of the extremes of a prehospital setting (e.g. cold, warm, dark, sunny, rain, snow). The evidence for non-physician performed thoracic ultrasound examinations differ regarding feasibility, quality, and accuracy and it is still debated if and how prehospital thoracic ultrasound should be implemented [14–16]. Only a few minor published studies explore the feasibility and accuracy of these ultrasound examinations.

The aim of this study was to 1) examine the feasibility and quality of prehospital thoracic ultrasound examinations performed by EMTs and PMs, and 2) to explore whether thoracic ultrasound leads to the EMTs and PMs changing initial patient management.

## Methods

### Study design and setting

The study is a retrospective quality-control study with prospective gathering of data. The study took place in a prehospital setting in the Region of Southern Denmark from November 2018 to April 2020.

The Danish healthcare system including the prehospital system is public and therefore available for free for all citizens. The prehospital setting in the Region of Southern Denmark consists of basic resource ambulances with two EMTs dispatched by an emergency medical dispatch center. The dispatcher can distribute the basic ambulance with different response times depending on the need, add on a paramedic, an anesthesiologist in a ground-based mobile emergency unit, or helicopter emergency medical service (HEMS) [7, 17].

There are 1,2 million citizens in Region of Southern Denmark distributed on 12.262 km^2^. In 2018, 39.501 ambulances were dispatched with a median response time of 8 min (interquartile range 6.0;11.0) [18]. The basic education of Danish EMTs is a 3-year education within the public health system. In the basic ambulances, one of the EMTs must, besides the mandatory basic education, have at least 12 months of supplemental education and experience. Paramedics have three years of practice as an EMT and additionally five weeks of theoretical and practical education and a final exam.

### Inclusion criteria

The Emergency Medical Services (EMS) personnel (covering EMTs or PMs) performed the ultrasound examinations on patients in their usual prehospital setting. Patients were eligible for inclusion in the study if they:

- Had made an emergency call for an ambulance due to respiratory symptoms or symptoms suggesting pathology in the lungs (e.g. pneumonia or pulmonary edema)
- Were assessed by the EMT or PM to be in a condition that allowed for transport to the hospital following normal traffic regulations (not applying horns and sirens)
- ≥ 18 years old

The study was conducted in conformity with the policy statement for the use of human subjects of the Declaration of Helsinki and was regarded as a quality assurance project by the Regional Committee for Medical and Health Research Ethics (registration number S-20182000-130). Approval for conducting the study as a quality assurance project was granted by the Prehospital Organisation in the Region of Southern Denmark (Journal no. 19/14433).

### Equipment and thoracic ultrasound protocol

**Fig. 1:**
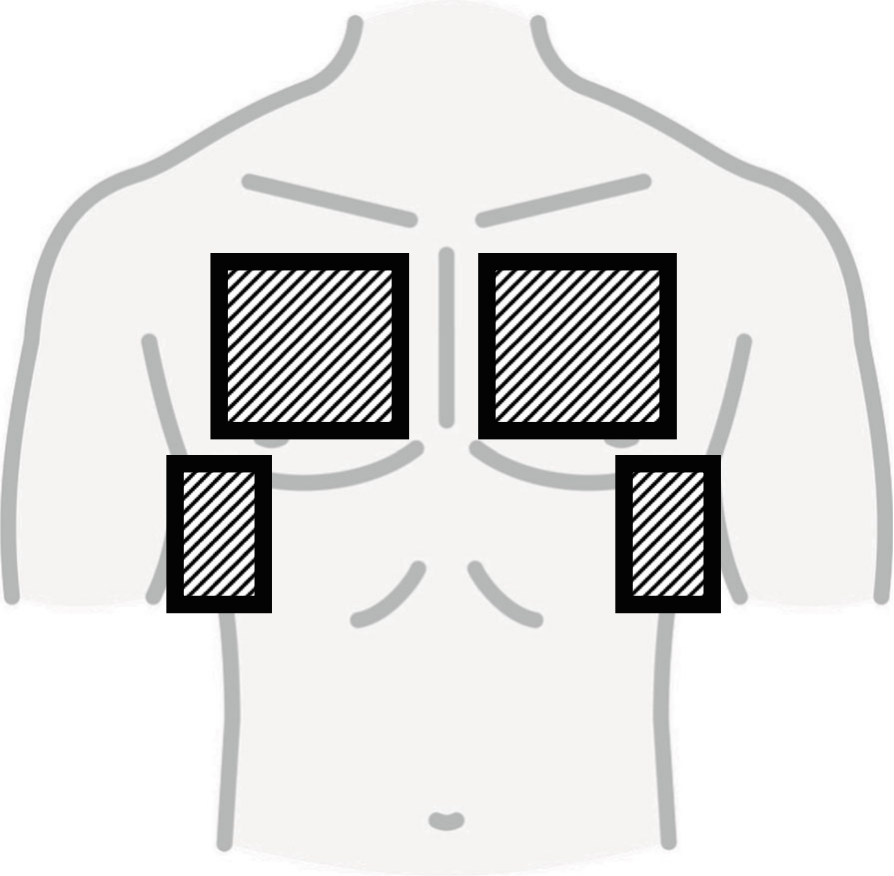
Graphical presentation of scanning zones

A portable ultrasound device with a low frequency, abdominal transducer (Lumify, by Philips) was used for training and during the study to obtain four standard views, Fig. 1. Two anterior views corresponding to the upper anterior zone in the BLUE protocol or zone 1 in the focused lung ultrasound (FLUS) protocol on the right and left hemithorax, and two lateral views corresponding to the posterolateral alveolar and/or pleural syndrome (PLAPS)-view of the BLUE protocol or zone 3 in the FLUS protocol on the right and left hemithorax [19, 20]. The ultrasonic findings considered to be pathological are presented in Table 1. These definitions are based on solid evidence in thoracic ultrasound [19, 21, 22].

**Table 1:** Sonographic pathologies and diagnoses

Sonographic finding	Diagnosis
More than two B-lines in more than two zones	Interstitial syndrome (e.g. cardiogenic pulmonary oedema, non-cardiogenic pulmonary oedema, viral pneumonia, acute respiratory distress, ect.)
More than two B-lines in two zones or less	Focal B-lines (e.g. pneumonia)
Lung point	Definite pneumothorax
Absence of lung sliding without recognition of lung point	Possible pneumothorax
Hypoeccoic or hypereccoic fluid with or without septation	Pleural effusion (simple or complex)
above diaphragm	
Consolidation	Larger: Pneumonia or atelectasis (compression or obstruction) Minor: pneumonia, atelectasis (compression or obstruction), peripheral pulmonary embolisms, malignancy
Thickened or fragmented pleura	Thickened pleural (e.g. following pleural inflammation or empyema, pleural plaques)

The table presents the sonographic pathological findings and how there are interpretated

Immediately after the patient was screened cf. the inclusion criteria and the four ultrasound clips obtained, the recorded ultrasound clips were marked with an identification number and transferred to a secure online platform. After transmission, the clips were deleted on the portable scanner to accommodate the requirements of the European Union general data protection regulations.

If the EMS personnel were able to prioritise with regard to their patient-related duties, a questionnaire about the ultrasound findings and clinical impact was filled out. The questions in the questionnaire were as follows;

- Was the image quality acceptable?
- Was interstitial syndrome present?
- Was a pneumothorax present or suspected?
- Was pleural effusion present?
- Did the ultrasound examination change the suspected diagnosis?
- Did the ultrasound examination change the treatment or management of the patient?
- Did the ultrasound examination lead to call the anesthesiologist in the ground-based mobile emergency unit?
- Did the ultrasound examination lead to changed visitation (transport to another department or institution)?

### Education and training

EMS personnel (*N* = 100) with more than one year of experience but no previous experience with ultrasound went through an educational program in focused thoracic ultrasound. The task of training all EMS personnel in the Region of Southern Denmark was considered impossible. The inclusion of EMS personnel was therefore restricted to EMS personnel operating in 24-h rotas in the five ambulance stations with the highest number of patient contacts.

The educational program covered a 4-h lecture including hands-on training. The instructors were physicians with expertise and research in thoracic ultrasound, as well as experience in the education of thoracic ultrasound.

The lecture covered; introduction to the ultrasound device and ultrasound in general, ultrasound physics, the normal focused thoracic ultrasound examination, and the pathological focused thoracic ultrasound examination.

For the purpose of this manuscript and potential future implementation of thoracic ultrasound in a prehospital setting performed by EMS personnel, a complete and comprehensive diagnostic thoracic ultrasound examination was not the aim. Therefore, only specific and selected parts of the possible diagnoses were included and considered relevant. These findings were based on solid evidence and consensus by the authors and experienced ultrasound operators involved in the project. The pathological ultrasound findings included; signs of pneumothorax, signs of interstitial syndrome, signs of pleural effusion. During the lecture, the EMS personnel had to train the practical and technical execution of the ultrasound examination on each other, and all personnel was to perform an ultrasound scan while being observed by the instructor to ensure proper execution and understanding.

### Assessment of the ultrasound clips and reference test

All files including ultrasound clips and questionnaires were exported to an institutional server with password-protected log-in. Assessment of the ultrasound examinations was carried out by an experienced thoracic ultrasound operator without knowledge of the prehospital sonographic findings. The assessor was also blinded to all patient information, patient history, co-morbidities, and symptoms, as well as which of the EMS personnel performed the scan. The clips were assessed twice;

- First, to determine image quality on a scale from 1 to 5 (1 being very low quality and unable to determine potential pathology, 5 being very high quality with images of the same quality as an experienced ultrasound operator would present),
- Second, to determine if pathology was present on the ultrasound clips

The ultrasound image quality was rated based on;

- correct depth and gain
- if two ribs were in a longitudinal axe (bat-sign) were present in the image and the pleural line horizontally
- if the transducer was kept still while recording
- if abdominal organs were present in lateral zones
- if there was a good overview in the picture

### Statistical analysis

Descriptive statistics including frequencies for the categorical variables are presented in number, percentage, and 95% Confidence Intervals based at binominal distribution. Cohen’s kappa was calculated to evaluate the interrater reliability between the EMTs and reviewer for the categories; normal scan (i.e. scan without interstitial syndrome, signs of pneumothorax, or pleural effusion), interstitial syndrome, signs of pneumothorax, and pleural effusion [23]. For interpretation of the kappa values following criteria were used (< 0.00) poor, (0.01–0.20) slight, (0.21–0.40) fair, (0.41–0.60) moderate, (0.61–0.80) substantial, (0.81–1.00) almost perfect [24]. Overall observed agreement (OA), specific positive agreement (SPA), and specific negative agreement (SNA) in percentages were calculated because these are regarded relevant as a more clinically relevant outcome measure. Specific positive agreement is calculated by following formula: SPA = 2a/(2a + b + c), while specific negative agreement by following formula: SNA = 2d/(2d + b + c) [25]. All statistics were performed using STATA 16.0 (StataCorp LLS, Texas, USA).

## Results

A total number of 631 thoracic ultrasound examinations were uploaded to the server during the 17 months study period. On average, each EMS personnel performed ≈6 scans (631 divided by 100 EMTs and PMs) but it was not possible to link each examination to a EMT or PM due to blinding. Thereby, it was not possible to examine to which extent each EMS personnel contributes to the ultrasound examinations.

Of the 631 uploaded examinations, 4 were excluded because they were tests of the transmission processes, 15 examinations were excluded because more than two of the four required ultrasound clips were uploaded as still pictures, and 22 examinations were excluded because they contained less than three clips. Therefore, a total of 590 examinations were included in the quality assessment and final analyses, corresponding to an upload feasibility of 93.5%. Twenty-eight examinations (4.8%) had only three ultrasound clips and were then rated based on three clips instead of four.

**Fig. 2:**
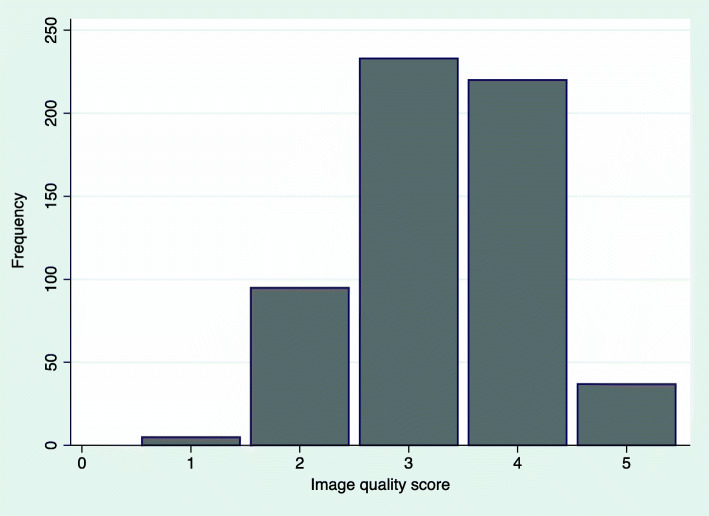
Image quality scores

The median image quality score was 3, IQ1 = 3 and IQ3 = 4, (mean 3.32, SD 0.85), and the distribution of the image quality scores are presented in Fig. 2.

Four-hundred-and-six examinations (68.5%) were by the reviewer interpreted as normal. In 74 cases (12.5%) the examinations were categorised as “normal but based on low-quality images” which potentially could have presented pathological findings if the image quality had been better.

Two significant errors were present in 77 examinations (13.1%); first that the ultrasound examination included clips from lateral zones which were scanned too caudally and no lung tissue were identified in the images (*n* = 37 examinations (6.3%)), and secondly, examinations included clips from the lateral zones which did not contain an abdominal organ (liver and spleen, respectively) so that pleural effusion could not be ruled out (*n* = 40 examinations (6.8%)).

The prevalence of the pathological findings established by the reviewer is presented in Table 2.

**Table 2:** Prevalence of findings in the 590 ultrasound examinations

Sonographic finding	number	%	(95% CI)
No pathological findings	404	68.5	(0.64–0.72)
Single B-line	87	14.8	(0.12–0.18)
Interstitial Syndrom (IS)	26	4.4	(0.03–0.06)
Focal B-lines	102	17.3	(0.14–0.20)
Pleural effusion	43	7.3	(0.05–0.09)
Right-sided	17	2.9	(0.02–0.05)
Left-sided	15	2.5	(0.01–0.04)
Bilateral	11	1.9	(0.01–0-03)
Pneumothorax			
Possible	5	0.9	(0.00–0.03)
Definite	0	0	(0.00–0.01)
Consolidation	72	12.2	(0.10–0.15)
Thickened or fragmented pleura	20	3.4	(0.02–0.05)
Free abdominal fluid (Ascites)	2	0.3	(0.00–0.01)
Enlarged spleen	1	0.2	(0.00–0.01)
Diaphragm with reduced motion	1	0.2	(0.00–0.01)
Pericardial effusion	1	0.2	(0.00–0.01)

Prevalence of the sonographic findings by the reviewer. Data are presented in number and percentage. Total number of ultrasound examinations were 590. Each scan could include more than one finding, e.g. left-sided pleural effusion recognised in left lateral zone and a single B-line in right anterior zone

Four-hundred-and-six questionnaires were completed by the EMTs and PMs, and were possible to merge with an ultrasound examination using an identification number. Kappa values and results of agreement between the reported findings by the EMS personnel and the reviewer are presented in Table 3.

**Table 3:** Diagnostic accuracy and agreement between the EMS personnel and blinded reviewer (*n* = 406 ultrasound examinations)

Diagnostic category	Frequency (=n)	Cohen’s kappa	Agreement
Normal^a^			
	EMS: 345	0.44	OA: 87.7%
	Reviewer: 365		SPA: 93.0%
	Both: 330		SNA: 51.0%
Interstitial syndrome			
	EMS: 42	0.26	OA: 89.9%
	Reviewer: 17		SPA: 30.5%
	Both: 9		SNA: 94.6%
Possible pneumothorax			
	EMS: 7	0.01	OA: 97.3%
	Reviewer: 4		SPA: 0%
	Both: 0		SNA: 98.6%
Pleural effusion			
	EMS: 24	0.69	OA: 96.3%
	Reviewer: 27		SPA: 70.6%
	Both: 18		SNA: 98.0%

Frequency, Cohen’s kappa value, and agreement in percentage. ^a^Normal = without interstitial syndrome, signs of pneumothorax, or pleural effusion. *OA* overall agreement, *SPA* specific positive agreement, *SNA* specific negative agreement

In 44 cases (10.8%), the EMS personnel reported that the ultrasound examination changed their initial suspected diagnosis, and it resulted in a change of treatment or management in 28 patients (6.9%). Four times (1.0%) the anesthesiologist in the ground-based mobile emergency unit were called and 39 patients were revisited to another department or institution other than originally planned (9.6%).

To summarise, the ultrasound examination entailed a change in treatment or visitation in 48 cases (11.7%) which in this study population encompasses a number-needed-to-scan of 8.5. The event rate was 0.118 (48 divided by 406) and as the event rate without the ultrasound examination was zero, on average (number needed to scan = 1/0.118 = 8.5) 8.5 patients should be scanned for one treatment or visitation to be changed. Change in treatment or visitation could be caused by both a pathological (inclusion of a pathology) or normal ultrasound examination (exclusion of pathology) depending on the initial suspected diagnosis.

When dividing the scans into two subgroups based on if the EMS personnel found the quality of the ultrasound examination sufficient or not, the mean image quality scores were 3.4 (SD 0.81, range 2–5) for the scans assessed as good quality versus 2.4 (SD 0.50, range 1–3) for the scans assessed as poor quality. This could indicate that the EMS personnel to some extent could assess whether their ultrasound examinations were sufficiently executed or not.

## Discussion

We sought to explore the feasibility and quality of EMS personnel performed focused thoracic ultrasound examinations. We found an overall acceptable image quality with only minor parts of the examinations deemed unacceptable or with quality too low for identifying sonographic pathology. Moreover, image quality scores differed with more than one point (3.4 versus 2.4, respectively), when dividing the examinations into two groups based on whether the EMS personnel defined the quality of the examination as good or poor. One could then argue, that the EMS personnel are aware of what defines a good and a bad thoracic ultrasound examination, and therefore are aware of their own competence and when to rely on the examination or not. Based on the proportion of successfully uploaded examinations and image quality, EMS personnel performed focused thoracic ultrasounds seems feasible.

There is a growing body of literature exploring the use, feasibility, and effect of prehospital ultrasound examinations [26, 27]. The papers suffer from significant heterogeneity, are often small studies, and differ in the personnel who perform the prehospital ultrasound examination as well as divergent protocols. In a majority of the cases, trained and experienced physicians in mobile emergency units are performing the examination. To our knowledge, few papers have been published pragmatically exploring EMS personnel’s ability to perform prehospital ultrasound examinations in a setting resembling everyday clinical practice [15, 28–40], and a minority of those focus on thoracic ultrasound or specific parts of a thoracic ultrasound examination, e.g. pneumothorax [15, 28, 29, 33, 35, 37, 39, 40]. Roline et al. present a study exploring non-physician performed ultrasound examination to rule-in or rule-out pneumothorax in a helicopter emergency medical service (HEMS) setting [40]. In this study, 54% of the ultrasound examinations were by an expert rated as “good quality” on a dichotomic variable (good vs. poor image quality). No other studies have rated image quality of thoracic ultrasound examinations on a broader scale and in a setting comparable the clinical everyday life.

It requires competent ultrasound operators to ensure acceptable image quality, interpretation of the findings, as well as the use of the findings to support clinical decision-making. The educational approaches and standards for EMTs or PMs in ultrasound have been discussed [41]. An educational program should include a theoretical part and a practical part so that the EMS personnel possess the ability to obtain the images, interpret the images, and can put the findings into context with other investigations (e.g. respiration rate, heart rate, and patient history). The literature, however, does not offer standardised assessment tools and evidence on proficiency levels for ensuring these skills. The EMS personnel in our study were trained by experienced physicians and the educational program included both theoretical and practical training but no summative or formative assessment was done [42]. This is a limitation to the study and the image quality scores could potentially increase if the educational program had strived for proficiency using a mastery learning approach including an assessment with solid validity evidence [43]. In a setting where ultrasound was to be implemented permanently in the ambulances, assessment and re-certification should be considered mandatory.

The benefit and patient-related outcome following an ultrasound examination is difficult to explore because many variables can affect the patient outcome – both pre- and intrahospital, e.g., time spent on the scene, initial treatment and management, choice of receiving facility, and transport time to the hospital. Therefore, the effect and clinical outcome is still a considerable and ongoing important debate [26]. In our study, the EMS personnel reported ultrasound resulting in a change in suspected diagnosis, treatment, management, or choice of receiving facility in 6–11% of the patients.

The aim of this study was not to investigate the clinical impact of EMT or PM performed prehospital thoracic ultrasound and we are not able to answer the question to which extent the scan can affect the clinical outcome. We did also not explore the time used at the scene and the time used at the ultrasound examination. This is a major limitation to the study since all decisions and management of a prehospital patient is a balance between quickly transportation to the hospital for advanced medical care and correct initial management. Our experience from an in-hospital setting reveals that inexperienced operators used 15–20 min on a complete thoracic ultrasound examination (14 zones) and thereby our assumption is that the 4-zone protocol can be done within 10 min [44].

More studies with a randomized, controlled set-up and in-hospital outcome measures are needed to explore the clinical effect and benefit of prehospital, EMS-performed thoracic ultrasound. However, when looking at the in-hospital evidence and prehospital physician-performed thoracic ultrasound, this examination could contribute to faster diagnosis and thereby initiation of correct treatment [26, 45, 46].

Extreme weather conditions could influence the feasibility and must be taken into account when implementing new prehospital strategies. In our study, only completed examinations were registered and transferred to the server. An unknown number of attempts of examinations that were not completed could potentially exist. Becker et al. explore these challenges for prehospital ultrasound and report equipment failure as the reason why the ultrasound scan was not performed in 25% of the cases (11 of 44 excluded patients) [15]. They conclude that only one of four pre-defined feasibility thresholds were met. Descriptive evaluation based on unstructured interviews with the EMS personnel did not reveal equipment failure as a significant issue and threat to the aim of our study.

An approach that is possible using the portable ultrasound device used in the study, is live transmission of the ultrasound scan to another portable device. This telemedical approach is feasible and deliberation with e.g., the anesthesiologist in the ground-based mobile emergency unit or the emergency medicine physician at the emergency department based on real-time transmission and interpretation of the images could support the EMS personnel’s decision-making and handling. Thus, some geographical or logistic challenges could be overcome in some remote areas. A concern in readily access to support and guidance from a physician through telemedicine, would be an overuse thus increasing costs of the system and potentially prolonging the time spent on scene. In our study, the EMS personnel, however, only needed support in 1% of the patients.

Since patients needing emergency treatment or transportation (e.g. severe respiratory failure) were excluded, the study results will undoubtedly reflect this selection bias. The study results are, however, probably a conservative estimate of the number of patients with pathological findings and thus potential clinical impact, since other studies generally have shown the greatest benefit of ultrasound in the most critically ill patients. Opposed to the conservative estimate of findings and clinical impact, the feasibility and image quality would probably have been negatively affected by including more critical patients in the study population. Thus, these results cannot necessarily be generalised to a population of more critical ill patients.

## Conclusion

In conclusion, it is possible for EMTs and PMs to perform focused thoracic ultrasound examinations with a high feasibility and image quality sufficient to determine if pathology is present or not. The EMS personnel’s assessment correlates to some extent with an experienced reviewer and their findings are most reliable for the inclusion of a normal scan or inclusion of pleural effusion. Implementation could possibly impact the number of patients receiving correct prehospital treatment and optimal choice of receiving facility.

## Data Availability

The datasets used and analysed during the current study are available from the corresponding author on reasonable request and if a data processor agreement is compiled and signed.

## Abbreviations

EMT: Emergency Medical Technician
EMS: Emergency Medical Service
PM: Paramedic
